# Delivering on the promise of competency based medical education – an institutional approach

**Published:** 2019-03-13

**Authors:** Damon Dagnone, Denise Stockley, Leslie Flynn, Rylan Egan, Richard van Wylick, Laura McEwan, Ross Walker, Richard Reznick

**Affiliations:** 1 Queen’s University, Ontario, Canada

## Abstract

The Royal College of Physicians and Surgeons of Canada (RCPSC) adopted a plan to transform, over a seven-year horizon (2014-2021), residency education across all specialties to competency-based medical education (CBME) curriculum models. The RCPSC plan recommended implementing a more responsive and accountable training model with four discrete stages of training, explicit, specialty specific entrustable professional activities, with associated milestones, and a programmatic approach to assessment across residency education. Embracing this vision, the leadership at Queen’s University (in Kingston, Ontario, Canada) applied for and was granted special permission by the RCPSC to embark on an accelerated institutional path. Over a three-year period, Queen’s took CBME from concept to reality through the development and implementation of a comprehensive strategic plan. This perspective paper describes Queen’s University’s approach of creating a shared institutional vision, outlines the process of developing a centralized CBME executive team and twenty-nine CBME program teams, and summarizes proactive measures to ensure program readiness for launch. In so doing, Queen’s created a community of support and CBME expertise that reinforces shared values including fostering co-production, cultivating responsive leadership, emphasizing diffusion of innovation, and adopting a systems-based approach to transformative change.

## Introduction

Among the numerous challenges of contemporary postgraduate medical education is the need to keep pace with rapidly evolving technology; the exponential growth of medical knowledge; patient safety initiatives that impact how physician training occurs; reductions in resident duty hours; threats to maintaining optimal continuity of care for patient; the renewed focus on trainee wellness; and the need to better accommodate learners’ prior knowledge and experience with more flexible educational models.^1^ However, in many ways we adhere to the early blueprint proposed in the Flexner Report – assuming it keeps pace with the times we live in over 100 years later.^2^

According to Frank et al., competency-based medical education (CBME) approaches have the potential to positively alter the future of medical training.^3^ In the Royal College of Physician and Surgeon’s (RCPSC) conception of CBME, these curriculum models de-emphasize time on task, instead focusing on the achievement of pre-determined entrustable professional activities (EPAs) and milestones that are the basis for learner progress and program completion.^4^ CBME is a fluid approach to education and allows for dynamic interactions between the needs of the learners and requirements of training programs. This approach ensures that capable residents will move through training at an individualized pace (faster or slower) in a more efficient manner, saving valuable resources, creating greater flexibility, and better preparing them for their individual paths to independent practice.^3^ The benefits of CBME may not be limited to trainees; the potential of CBME extends to all stakeholder groups.

Within Canada, the College of Family Medicine of Canada (CFPC), responsible for the accreditation of Family Medicine programs, initiated their transition to CBME in 2010.^5^ Embracing this challenge, the Queen’s Family Medicine program operationalized an approach to CBME that exceeded the requirements of the CFPC, and provided guidance to the faculty-wide initiative (Schultz, 2016).^6^ The Queen’s approach, launched in 2010, required enhanced approaches to curricular planning, assessment documentation, and the use of EPAs.^7^

Later in 2014, the RCPSC, the body responsible for the accreditation of all other graduate medical education programs, unveiled the Competency by Design (CBD) project and mapped out a seven-year transition for all postgraduate medical education specialty training programs across Canada. The RCPSC recommended implementing a more responsive and accountable training model with four discrete stages of training, explicit specialty-specific EPAs with associated milestones, and a programmatic approach to assessment across residency training programs^8,^^9^ Embracing the potential of CBME, and with permission from the RCPSC, Queen’s University set a goal to be the first university in Canada to implement CBME across all of its twenty-nine specialty programs starting with the incoming cohort (n=93 trainees) in July 2017. It was a seminal decision to move all our postgraduate medical education programs in line with our Family Medicine program at the same time, rather than by the national specialty cohort design proposed by the RCPSC. This decision meant the entire system at Queen’s had to evolve in tandem to ensure readiness for CBME.

Unique to its university environment, many circumstances seemed advantageous for Queen’s to make the transition: a relatively small institutional size, a collegial atmosphere, a centralized funding formula for teaching faculty, specialized assessment expertise, outstanding information technology resources, a state of the art clinical simulation centre, colleagues from the Department of Family Medicine who had successfully implemented competency-based education previously, and a shared educational vision from institutional leadership.

Embarking on this ambitious path required a comprehensive implementation strategy that could leverage the numerous institutional strengths, engage all partners and key stakeholder groups in the change management process, build a central CBME governance structure, and promote a shared leadership model across twenty-nine postgraduate programs. Over a three-year period, Queen’s University School of Medicine navigated a systems-based approach to transformative change and launched CBME curricular models in residency education across all training programs on July 1, 2017.

## Creating a central team

Beginning in 2014, the decanal leaders within the School of Medicine at Queen’s University set the foundation for CBME implementation. Key to this foundation was clearly articulating, in the School of Medicine’s strategy, that a collective goal was the design and implementation of new educational models. As such, delivering on this strategy was a shared responsibility of the decanal team and the School’s departmental leadership teams. The School of Medicine and its system-wide physician practice plan decided to co-invest in one-time start-up costs that provided each of the twenty-nine programs with the equivalent of one day per week protected time for a faculty member and the ability of each program to hire part-time education expertise. In addition, the School of Medicine provided significant in-kind support for information technology (IT) infrastructure, educational consultancy, and leadership salaries.

**Figure 1:**
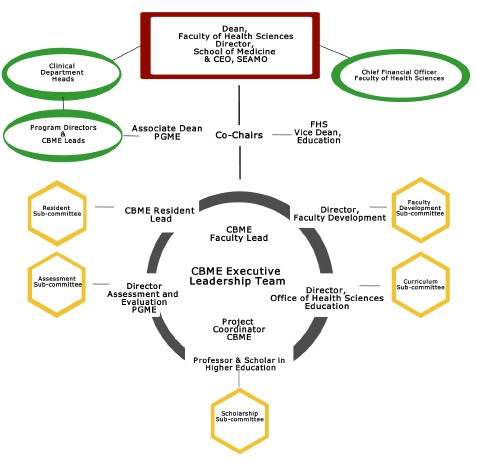
Queen’s CBME Governance Model

Project governance was established to set operating boundaries for the CBME project through the appointed CBME Executive Team (Figure 1). The executive team’s mandate was to define the project scope and identify operating aspects and relationships of the implementation. The executive team consisted of faculty (MDs & PhDs), a project coordinator, a resident leader, and staff who cumulatively had expertise in the areas of leadership, curriculum, assessment, faculty development, scholarship, and project management. The CBME executive team acted as the governing body and set project deliverables, approved project milestones, and worked together coordinating various CBME sub-committees to ensure each piece of the CBME project implementation was executed, on time, and on budget. The CBME executive team was directly accountable to the Dean of the Faculty of Health Sciences and regularly interacted with the department heads and program educational leaders.

Beginning in 2015 and through to 2017, the CBME executive team carried out important foundational work. The first task was the submission and subsequent approval of the RCPSC Fundamental Innovations of Residency Education (FIRE) proposal.^10^ This process was essential because deviating from the existing national accreditation standards and implementing new CBME curriculum models across twenty-nine specialty programs at Queen’s required the permission from the postgraduate accreditation body in Canada – the Royal College of Physicians and Surgeons of Canada. From there, key priorities included:

1. Development of an CBME Executive Team governance model
2. Creation of guiding principles for CBME implementation
3. Development of an institutional program evaluation initiative
4. Creation of a diverse stakeholder engagement process
5. Provide supporting expertise and collaborative processes for the co-creation of stage-specific EPAs for all specialty programs.
6. Creation of multiple institutional support systems for curricular reform, assessment reform, faculty development, simulation resource expansion, and medical education scholarship
7. Creation of a comprehensive CBME budget model
8. Approval for fundamental changes to the faculty’s accountability framework as it relates to the transition and sustainability of CBME across all postgraduate programs at Queen’s University

## Roles of sub-committees

Meeting for over two years on a weekly basis, the CBME executive team was supported by five CBME sub-committees (Curriculum, Assessment, Faculty Development, Scholarship, and Resident Leadership) that were led by members of the executive team. The sub-committees were comprised of faculty (MD & PhD), residents, and education consultants (MEd or PhD education specialists). These sub-committees provided regular direction and feedback to the central team that was integrated into the overall project implementation plan. The following provides an overview of each committee and their purpose within our CBME implementation.

### Curriculum

The Curriculum Sub-committee was established to provide a forum for program leaders to discuss methods of curricular reform within postgraduate medical education. The Curriculum Sub-committee provides leadership to the CBME project team in regard to best practices, policies and protocols to aid with Queen’s transition to a competency-based medical education model. This group acts in an advisory capacity and in consultation with the CBME Executive Leadership Team.

The goals of the Curriculum Sub-committee were:

- Development and support of tools to assist specialty programs in aligning curriculum activities to EPAs within and across stages
- Develop a preparation course that supports all trainees for the Medical College of Canada Licensing Exam - Part II
- Contribute to the design of CBME workshops, specifically with regard to curricular components (required training experiences, rotations, and document creation).

### Assessment

The Assessment Sub-committee was established as a collaborative working group to address assessment priorities related to Queen’s transition to a CBME model. Group membership was purposefully designed to leverage input from a wide range of specialties and sub-specialties, draw on assessment expertise from across the institution (e.g., Centre for Teaching and Learning, Faculty of Education), and facilitate access to the Education Technology Unit responsible for CBME module development in Elentra™. Initially, members of this committee focused on developing the Queen’s constructive alignment curriculum design process. Employing a backwards design orientation, a template to guide program development of EPAs, assignment of enabling competencies/milestones, designation of required training experiences, and determination of assessment requirements was developed. Once CBME program development was underway, attention of the committee shifted to an advisory capacity, in consultation with the CBME executive leadership team. Priority responsibilities for the group were defined as:

- Defining practice guidelines to support the development and evaluation of CBME programmatic assessment, guided by the principles of sound assessment practice.
- Providing in-put on the design of Elentra functionalities.
- Supporting the sharing of assessment innovation across programs.
- Establishing assessment-blueprinting strategies.
- Contributing to the refinement of CBME assessment policy.
- Supporting faculty development initiatives related to assessment.

### Faculty Development

The Faculty Development Sub-committee was established to support the implementation of CBME at Queen’s through the development of program leaders and front-line faculty. Over a two-year period, the design and execution of a multi-faceted strategy for CBME program leader and frontline faculty development included: single and multi-day program leader workshops, regular small group sessions, seminars, webinars, on-line resources, regional teaching site orientation sessions, and weekly one-on-one consultations with expert members from the executive team.

In the process, faculty leaders were required not only to become familiar with the tenants of CBME, but also needed to create new curricular documents for their individual programs such as stage-specific EPAs with milestones and develop stage-specific assessment plans that included novel assessment tools. This work was performed throughout the academic year, usingone-on-one consultative meetings with experts to meet the iterative and evolving needs of the faculty leaders, and through strategically planned collaborative workshops. Thereby all program leaders came together to talk about best principles in a shared leadership environment to create program specific documents defined by stage-specific EPAs. Most importantly, the series of multiple workshops over a period of more than two years provided a forum for all program leaders to engage in a community of shared learning and leadership, whereby collaboration and shared experiences greatly enhanced the momentum and acceptance of the shift to CBME.

### Resident Leadership

The CBME Resident Sub-committee was established to provide a forum for discussion and advocacy concerning resident issues in anticipation of the transition and implementation of CBME. The group provides support to the CBME executive team in developing effective and transparent communication strategies to inform current and incoming residents of CBME expectations and updates. The group also communicates closely with the Resident Doctors of Canada and the Professional Association of Residents of Ontario regarding CBME implementation at Queen’s, and represents the interest of residents at the hospital, provincial, and national levels during the CBME transition at Queen’s.

### Scholarship

The Scholarship Sub-committee was established for the purpose of leveraging the expertise of academic researchers and scholars within the Faculty of Health Sciences. The Scholarship Sub-committee provides leadership to the CBME project team related to research-based best practices, policies and protocols to aid with Queen’s transition to a CBME model. Its mandate is to review measures required to strengthen, enhance and promote the output of scholarly activity relating to Queen’s School of Medicine’s transition to a competency-based medical education model. Its goals are to:

- Contribute to the design of ongoing faculty development workshops, specifically with regard to academic scholarship
- Direct the allocation of grant money and supported projects
- Contribute to the development of a systematic institutional support structure for the development, implementation, and sustainability of CBME scholarly activities

## Additional areas of foci

In addition to the five key executive sub-committees, four additional areas of focus were essential to the central plan of CBME institutional implementation: education technology, simulation capacity, stakeholder communications, and program evaluation.

### Education technology

The education technology steering committee was assembled to oversee development of the electronic resident portfolio (CBME module), an extension of the integrated teaching and learning platform Elentra^**TM**^.^11^ Led by the manager of the Education Technology Unit, along with key members of the executive team, an iterative process of design, piloting, and feedback was created to ensure essential design elements required for the implementation of CBME were addressed.

The Elentra CBME module includes data collection, aggregation, and advanced display functionality and is customizable to differential program needs. Programs have the freedom to select from and customize a variety of assessment templates and tag assessments to any level of the curriculum hierarchy (e.g., EPAs, CanMEDS Roles, key and enabling competencies, and milestones).

Once published, assessments can be triggered by residents and faculty at the bedside or scheduled for distribution by a program. Assessment information gathered from multiple assessors (e.g., attending physicians, multidisciplinary health care team members, patients, junior learners) across various clinical and academic settings, and over time, are combined and displayed on the “resident dashboard” which is accessible to the resident, their program director, academic advisor, and the program competence committee.

The dashboard supports residents and faculty to monitor performance in real time and track progress over time. Advanced filtering functionality allows users to easily search resident assessment data to inform progress and promotion decisions. Reporting functionality permits academic advisors to record performance review meetings and upload summaries for review by individual program leaders. Additional reporting functionality permits program leader sign-off EPAs on the dashboard interface.

### Stakeholder engagement

Essential to the project’s change strategy was regular and ongoing stakeholder and partner communication. With such an ambitious project, it was critical to inform and receive feedback from the many interconnected groups involved in postgraduate medical education. This feedback occurred in many forms on a regular monthly basis through one-on-one meetings, teleconferences, email communications, newsletters, committee meetings, and regional and national presentations.

Stakeholders fall into into numerous overlapping groups that include many CBME sub-committee members, hospital partners at our University and distributed sites, frontline faculty, current resident trainees, program leaders, decanal leaders, patient advisors and community members, and the RCPSC executive leadership.

The goals for our stakeholder group were:

- Build and sustain the momentum of support for the institutional change to a competency-based medical education curriculum;
- Mitigate resistance to change, both internally and externally;
- Foster effective communications to all stakeholders; and
- Enhance Queen’s School of Medicine’s brand by ensuring the CBME transition aligns with the School’s mission to advance the science and practice of medicine to benefit the health and well-being of the population while doing this through excellence in education, care and research.

### Simulation capacity

The introduction of CBME across all postgraduate medical education programs requires significant resources and a strategic vision for skill acquisition, training, and assessment at the Queen’s Clinical Simulation Centre (CSC) and the Department of Biological and Molecular Sciences (DBMS) Anatomy Lab.

At present, the CSC and DBMS Anatomy Lab are responsible for providing simulation-based learning opportunities for all UGME and PGME trainees. The implementation of CBME across all of our graduate medical education programs requires additional resources to support our educational mandate, both in curriculum and assessment. This includes additional simulation equipment, supplies, simulation technician time, standardized actors, video equipment capabilities, task trainers, and cadaver specimens. Faculty support and faculty development must also occur to ensure faculty teachers develop the prerequisite skills required to lead newly created training experiences and enhanced competency assessment in simulation environments. As a result, additional funding was allocated to meet the needs of the new CBME curricula.

### CBME program evaluation

Starting in 2015 at the beginning of the CBME project, an institutional program evaluation strategy was initiated to document the many stages of the change process. The key methods being used for the evaluation of the initial CBME implementation are the Concerns Based Adoption Model (CBAM) and Outcome Harvesting. CBAM is based on three components: Stages of Concern questionnaires, Levels of Use interviews, and an Innovation Configuration map.^12^ The components are overlapping elements which, when taken together, can inform the process of change across an institution. In CBAM, change is described as a process encompassing three stages: creating the foundation, implementation, and ensuring sustainability. The strengths of this approach allow for the identification of stakeholder groups or specialty programs that may be struggling with the transition, and identifies needed supports early in the implementation process. Ongoing monitoring of CBME will continue using Outcome Harvesting.^13^ Outcome Harvesting is suitable for evaluating complex programming contexts and is especially useful when the aim is to understand how individual outcomes contribute to broader system-wide changes. This approach draws on the knowledge of key informants who understand the change that has taken place, as well as their contributions to that change. Overall, this comprehensive approach to capturing data across the institution will help inform the future directions and central support required for the CBME project.

In addition to the comprehensive data captured from all stakeholder groups at an institutional level, individual programs have embarked on their own “program specific” program evaluation processes. At the program level, the shift to CBME represents many changes and iterative processes in action. Using rapid cycle evaluation models^14^ and other approaches to program evaluation, all programs will be supported to complete ongoing program evaluation as the CBME implementation process continues to unfold.

The preliminary data associated with launching CBME at Queen’s University reveals that all 29 specialty programs (100%) launched their incoming resident cohorts into CBME training programs as planned with stage-specific EPAs and accompanying milestones adapted from the CanMEDS framework. All residents and frontline faculty have access to the electronic assessment platform (Elentra) and ongoing efforts are being made to reach out to all frontline faculty using “just in time” methods, as well as, formal teaching sessions within programs for ongoing faculty development. According to preliminary data, CBME assessment is on track with the estimated mean number of completed CBME assessments per month, per resident rising from five in September 2017 to six in February 2018. Apart from significantly reducing the amount of time spent away from residents’ home programs in the initial stages of training, Curricular reform has been minimally effected in the first year of implementation. However, this will be an area of focus in ongoing implementation to ensure proper curricular changes accompany the vision of CBME.

## The creation of twenty-nine program teams

We identified the need for additional CBME champions to assist in leading program preparation and implementation of the CBME transition. This was identified as high priority and changes were made to the funding structure of postgraduate medical education to support the new CBME educational deliverables. At the outset of the project in 2015, the central CBME executive team understood that the scope of CBME implementation across twenty-nine specialty programs would require additional leadership within each program. This occurred initially with the creation of twenty-nine program CBME Leads and was instituted centrally across our funding framework. The title of CBME lead brought with it academic protected time (0.1-0.2 FTE) and a formal role description, for an additional faculty member in each program. The CBME Lead works collaboratively with the existing program director for that specialty.

With support from the academic funding formula of the School of Medicine, education consultants were hired to provide program support. Their responsibilities included, but were not limited to curricular document creation, programmatic assessment development, facilitating frontline faculty training, and integrating EPAs and assessments into the Elentra platform. Over time, more than 80% of all programs had an education consultant on the program team. Most education consultants work across multiple programs and meet regularly as a group to promote the cross-pollination of CBME innovation. This distributed network of educational expertise is a powerful resource that supports CBME leads to operationalize CBME.

One year prior to the institutional CBME launch, resident leads from each program were recruited to assist with preparation, readiness, and implementation by working alongside the program directors, program CBME leads, and educational consultants. In addition, the resident CBME leads were vital in the communication and promotion of CBME for residency selection within their programs and advocacy for the benefits that CBME could provide. After meeting monthly for six months, the co-chair of the Resident sub-committee was invited to become a permanent member of the CBME executive team and the critical role of resident leadership was well recognized within the program teams.

**Figure 2:**
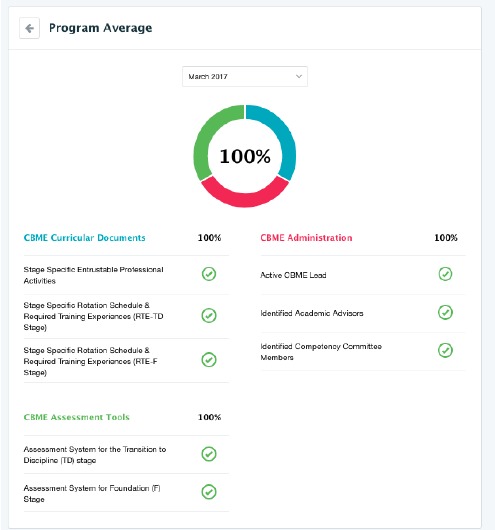
CBME readiness dashboard

Overall, the creation of twenty-nine program teams made up of program directors, program CBME leads, educational consultants, program resident leads, and program administrators were essential to the successful implementation of CBME. After two years of preparation, and in partnership with existing resident program committees, the accelerated path to CBME implementation had become a reality through shared work, strategic planning, central executive team support, and organic collaboration. A centralized electronic dashboard was created to ensure all programs had achieved all necessary curricular, assessment, and administrative objectives three months prior to the July 1^st^ timeline launch (Figure 2).

In the six months before the July 1^st^, 2017 launch deadline, the level and intensity of central support provided by the CBME executive team intensified. It was essential that the CBME leaders from each program, as described above, were provided with the necessary tools, resources, and desired one-on-one consultations to customize their implementation plans and overcome any remaining hurdles/barriers. Multiple central “just in time” initiatives were utilized specifically addressing the needs of the frontline faculty, program academic advisors, and the new members of each program’s competence committee. These initiatives included multiple targeted faculty development workshops and training sessions at local and regional hospital sites, customized program level frontline faculty and resident leader training, online training modules and guides, one-on-one individualized training sessions with program leaders, and a dedicated central daytime drop-in space (“The CBME Central Hub”) staffed with project leadership for a four-week period before and two-week period post-launch.

### Lessons learned

The institutional approach taken to implement competency-based medical education has been both rewarding and challenging. Reflecting on the greatest lessons learned over the first three years of the project, the following would be deemed most important to the journey:

1. Creating numerous formal and informal opportunities for program leaders to collaborate regularly in small and large group settings has helped created a unique community of engaged medical education leaders. Delivering cross-specialty program leader workshops to co-create EPAs, milestones, novel assessment tools, and drive end-user development of our CBME electronic platform (Elentra) has been the most valued approach we undertook.
2. Change management theory has provided a fundamental guide to the institutional plan. Concepts such as the diffusion of innovation allowed programs to progress through the CBME transition at their own pace, while being supported centrally to achieve baseline outcomes. Other principles such as early piloting of EPAs and assessment tools, ongoing stakeholder engagement using existing meeting forums, with resident leaders and supporting early scholarship work have been critical to the strategic planning process.
3. CBME theory, including EPAs, milestones, programmatic assessment, and entrustment scores, involve many new concepts that require time for faculty leaders to fully grasp. In truth, understanding of these concepts evolves over time and the explicit acknowledgement of this emergent understanding must continue to occur. This is supported with program specific “just in time” faculty development and resident development sessions, as well as understanding gained through program evaluation processes.
4. Everything is inter-connected within an academic health sciences center. Early on it was apparent that changes in any and all programs have an institutional impact. This relates to learner training, faculty needs, rotation schedules, ongoing patient care, administrative resources required, and leadership frameworks among other things. It is essential to keep the delivery of excellent patient and family centered care at the forefront of the CBME initiative and can be achieved with thoughtful integration of CBME educational initiatives into the clinical work flow across the many dynamic clinical settings.
5. Engaging all stakeholders in regular and meaningful ways is a challenge, even when it is recognized as a high priority. Effective communication requires a substantial investment of time and comes in many forms. There is no substitute for keeping key enablers of change informed – both for their support and managing resistance to forward progress. As well, a unified message is paramount and must evolve as it incorporates stakeholder feedback into the strategic plan. This was achieved by integrating brief but frequent CBME touch point discussions during leadership meetings, academic teaching rounds, and protecting time for urgently evolving communication needs. The weekly CBME executive team meetings ensured that the principal leaders were kept abreast of emergent issues and remained responsive to the evolving nature of the transition.
6. Building a reliable, effective, and forward-thinking education technology platform (Elentra^®^) was also essential. This required ongoing leadership and collaboration to ensure all CBME project needs were being captured in the build process with the functionality maximally benefitting end users – residents, frontline faculty, academic advisors, and competence committees. Having a diverse group of experts involved in the design and development process (technology, education, and clinical) ensured a strong, user-friendly platform.

### Ongoing opportunities and challenges

1. Continuing to support all program leaders during CBME implementation requires ongoing central resources and coordination of numerous iterative processes designed to make improvements. Managing the required changes, on-going quality improvements, and adapting to unanticipated consequences of CBME will require a continuous commitment of resources (time, money, expertise) to this multi-year project.
2. The CBME transition at Queen’s University is running concurrently with the Royal College of Physician and Surgeons of Canada (RCPSC) Competency by Design (CBD) project. Over time, all programs at Queen’s will transition with their specialty committees across the country to a national set of stage-specific EPAs and new training requirements within each program. This will demand a coordinated and careful approach so that no resident is adversely affected.
3. Curricular reform and pursuing a more time-independent path to training brings with it many logistical challenges. Postgraduate medical trainees are learners within the university and are also patient care service providers paid by the hospitals. Designing a new training paradigm that provides more flexibility in clinical rotations (type, duration, location, service provisions) will be an ongoing experiment that will require patience.
4. Developing an electronic platform to support all users to document, collate, track and monitor end user data for curricular, assessment, and evaluation purposes demands a process of iterative system development. This process must be integrated into the workflow of the development team and carefully managed with respect to prioritizing development decisions that provide both program specific enhancements, while at the same time delivering the greatest benefit to all users across all programs.

### Conclusion

With the CBME launch on July 1, 2017 and moving towards our next phase of CBME implementation at Queen’s University, the decanal leadership and CBME executive team will remain focused on ensuring that CBME is implemented as intended over the coming years. The ongoing engagement and collaborative contributions from key stakeholders will be essential to continue our journey towards meaningful transformative change in postgraduate medical education. There will also be unexpected challenges, and possibly unintended outcomes, with the adoption of this innovation. It will be imperative that we accumulate evidence of the impact of CBME and continue to approach implementation as an iterative process committed to programmatic quality improvement, informed by on-going program evaluation, and ultimately resulting in improved outcomes for our patients.
